# Liposomes as Carriers of GHK-Cu Tripeptide for Cosmetic Application

**DOI:** 10.3390/pharmaceutics15102485

**Published:** 2023-10-18

**Authors:** Michał Dymek, Karolina Olechowska, Katarzyna Hąc-Wydro, Elżbieta Sikora

**Affiliations:** 1 Faculty of Chemical Engineering and Technology, Cracow University of Technology, Warszawska 24, 31-155 Kraków, Poland; michal.dymek@doktorant.pk.edu.pl; 2 Faculty of Chemistry, Jagiellonian University, Gronostajowa 2, 30-387 Kraków, Poland; karolina.weder@uj.edu.pl (K.O.); hac@chemia.uj.edu.pl (K.H.-W.)

**Keywords:** liposomes, copper tripeptide, encapsulation efficiency, cosmetics

## Abstract

Liposomes are self-assembled spherical systems composed of amphiphilic phospholipids. They can be used as carriers of both hydrophobic and hydrophilic substances, such as the anti-aging and wound-healing copper-binding peptide, GHK-Cu (glycyl-L-histidyl-L-lysine). Anionic (AL) and cationic (CL) hydrogenated lecithin-based liposomes were obtained as GHK-Cu skin delivery systems using the thin-film hydration method combined with freeze–thaw cycles and the extrusion process. The influence of total lipid content, lipid composition and GHK-Cu concentration on the physicochemical properties of liposomes was studied. The lipid bilayer fluidity and the peptide encapsulation efficiency (*EE*) were also determined. Moreover, in vitro assays of tyrosinase and elastase inhibition were performed. Stable GHK-Cu-loaded liposome systems of small sizes (approx. 100 nm) were obtained. The bilayer fluidity was higher in the case of cationic liposomes. As the best carriers, 25 mg/cm^3^ CL and AL hydrated with 0.5 mg/cm^3^ GHK-Cu were selected with *EE* of 31.7 ± 0.9% and 20.0 ± 2.8%, respectively. The obtained results confirmed that the liposomes can be used as carriers for biomimetic peptides such as copper-binding peptide and that the GHK-Cu did not significantly affect the tyrosinase activity but led to 48.90 ± 2.50% elastase inhibition, thus reducing the rate of elastin degeneration and supporting the structural integrity of the skin.

## 1. Introduction

Biomimetic peptides are a group of very effective biocompatible raw materials, consisting of short chain amino acid sequences. They may be of synthetic or natural origin and can even be isolated from venomous animals [1]. According to the mechanism of the activity of the peptides, they can be divided into several groups: signal peptides—stimulating cellular processes of collagen production increase; carrier peptides—delivering trace elements, such as copper or manganese, involved in enzymatic reactions; and neurotransmitter-affecting peptides—reduce wrinkles via their Botox-like muscular relaxation [2,3,4,5]. One of the most popular peptides, currently widely used in skin and hair products is copper-binding tripeptide (GHK-Cu) [6,7]. It is a highly hydrophilic peptide that consists of three amino acids: glycine, histidine and lysine (Gly-His-Lys or GHK), with copper cations binding ability. GHK tripeptide has been found in human serum and plasma [8,9]. The amount of copper found ex vivo in human stratum corneum and the epidermis itself has been considered to be significant [10]. Copper tripeptide is capable of skin penetration and acting as a delivery system for metal cations, and it can be used in patch technology for anti-inflammatory therapy. GHK peptide as a complexing agent for copper ions increases the permeation rates of Cu^2+^, presumably accelerating the migration of copper ions through lipophilic stratum corneum [11]. GHK-Cu is known for lowering the healing time of damaged skin; it is capable of modulating matrix metalloproteinases expressions that are crucial for the healing process [12]. Moreover, the tripeptide increases collagen, elastin and glycosaminoglycan synthesis and supports the function of dermal fibroblasts [13]. The collagen IV synthesis may also be increased through the use of the GHK-Cu and hyaluronic acid (HA) combination [14].

Since peptides are hydrophilic molecules; they have difficulty crossing through the external skin layer (stratum corneum) [15]. In order to improve the permeability of molecules and thereby increase the therapeutic effect of the cooper binding tripeptide, as well as other hydrophilic peptides, various techniques can be used [16,17]. The modification of the GHK peptide behaviour by changing its polarity is achieved through the addition of hydrophobic acyl moieties, e.g., hexanoyl, myristoyl or decanoyl [18]. The palmitoyl form of GHK is frequently used in anti-aging cosmetics [19]. Moreover, to improve permeability of the active ingredients, their encapsulation in delivery systems such nanoemulsions, solid lipid nanoparticles, nanostructured lipid carriers and nanocapsules is used [20]. Currently, one of the most popular skin-delivery systems are liposomes, which are spherical carriers consisting of an aqueous core surrounded by a phospholipid bilayer. Liposomes are biocompatible, biodegradable, non-toxic and non-immunogenic carriers, and they are both hydrophilic and hydrophobic by nature [21,22,23,24,25]. An additional advantage of these phospholipid vesicles is the possibility of the structural modification of their membranes using sterols [26], polymers [27], proteins [28] or sugars [29]. The liposomal systems can be prepared by various methods, including thin-film hydration [30], microfluidics [31], reverse phase evaporation [32] and ethanol injection [33].

In the case of GHK-Cu, only two studies deal with the encapsulation of the tripeptide in liposomes [34,35]. S. Erdem et al. [34] studied the properties of GHK-Cu-loaded liposomes. They applied a thin-film hydration (TFH) method with subsequent sonication to obtain the lipid carriers based on dipalmitoylphosphatidylcholine (DPPC) or soybean lecithin. X. Wang et al. also used the lipid film hydration method, supported by ultrasonic homogenization and extrusion to prepare liposomes [35]. However, their research showed that the peptide encapsulation efficiency was not high, and the role of various factors on the efficiency of peptide encapsulation was not sufficiently explained.

In our work, the potential use of both cationic and anionic hydrogenated lecithin-based liposomes as carriers of GHK-Cu tripeptide was studied. The influence of the qualitative and quantitative composition of lipids and GHK-Cu concentration on the liposome properties were investigated. The liposomes were prepared using the thin-film hydration method but were combined with freeze–thaw cycles and the extrusion process.

## 2. Materials and Methods

### 2.1. Materials

The copper tripeptide (SpecPed-GCu11P, Spec-Chem Industry Inc., Nanjing, China) was of cosmetic grade and was kindly supplied by Alfa Sagittarius (Kraków, Poland). Hydrogenated lecithin (Emulmetik 950, Lucas Meyer Cosmetics, Massy, France) was kindly supplied by Naturallia Sp. z o.o. (Brzeg, Poland). Dicetyl phosphate, N-Succinyl-Ala-Ala-Ala-p-nitroanilide, elastase from porcine pancreas, mushroom tyrosinase and L-DOPA were all purchased from Sigma-Aldrich (Merck KGaA, Darmstadt, Germany). Stearylamine (97%) and cholesterol (95%) were purchased from Alfa Aesar (Thermo Fisher (Kandel) GmbH, Kandel, Germany). From Pol-Aura (Zawroty, Poland), 0.1 M Tris-HCl buffer was purchased. All other chemicals, such as potassium dihydrogen phosphate (POCh, Gliwice, Poland), sodium hydrogen phosphate (POCh), methanol (Chempur, Piekary Śląskie, Poland) and acetonitrile (Chempur), were of analytical grade. Spectra/Por 1 regenerated cellulose membrane with a 6–8 kDa molecular weight cut-off (MWCO), which was used in the microdialysis tests, was purchased from Spectrum Laboratories Inc., (DG Breda, The Netherlands). The deionized water used in all formulations was additionally filtered through a Milli-Q system.

### 2.2. Preparation of Empty and GHK-Cu-Loaded Liposomes

Liposomes were prepared using the thin-film hydration method. Appropriate amounts of lipids (lecithin, cholesterol and dicetyl phosphate (DCP) or stearylamine (SA) for anionic and cationic carriers, respectively) were dissolved in chloroform. The solvent was then removed using a rotary evaporator under reduced pressure at 45 °C and at 60 RPM for 20 min. The resulting lipid film was hydrated with a phosphate buffer solution (PBS) at pH = 6.00 for empty liposomes or with a GHK-Cu solution in the PBS at various concentrations (1 mg/cm^3^, 5 mg/cm^3^ or 10 mg/cm^3^) for peptide-loaded liposomes. The total amount of the hydrating solution was added to the lipid film, and the process was carried out for 5 min at 260 RPM in a water bath (70 °C). The total lipid concentration in the prepared formulations was 10, 25 or 50 mg/cm^3^. Cholesterol was used to potentially reduce the solutes’ permeability through the lipid bilayer. The lecithin-to-cholesterol weight ratio was kept constant at 14:1 for all samples in order to obtain high stability of the liposomes [36]. The obtained crude liposomes were subjected to five freeze–thaw cycles in liquid nitrogen and then extruded six times (twelve passes) through a polycarbonate membrane with 100-nm pore diameter using a hand-held Avanti mini extruder (Avanti Polar Lipids Inc. Alabaster, AL, USA). The liposomes were then stored at room temperature. The compositions of the prepared anionic (AL) and cationic (CL) formulations are shown in Table 1.

### 2.3. The Physicochemical Properties of Liposomes

#### 2.3.1. Size Analysis of Prepared Liposomes

The hydrodynamic diameter and size distribution, expressed as the polydispersity index (PDI) of the prepared vesicles, was measured using the dynamic light scattering (DLS) method by Malvern Zetasizer Nano ZS apparatus (Malvern Panalytical Ltd., Malvern, UK). The Helmholtz–Smoluchowski equation was used for the calculation of the zeta potential. The measurements were performed at 25 °C and repeated three times for each sample. All of these parameters were tested over a period of four weeks every seven days to determine the stability of the liposomes. During the stability tests, liposomes were stored at room temperature (20–25 °C). The temperature conditions were chosen due to the potential cosmetic application of liposomes, which requires their stability at room temperature [37].

#### 2.3.2. Transmission Electron Microscopy (TEM) Imaging of Liposomes

TEM analysis of the liposomes was conducted in order to confirm their size and shape. A volume of 5 µL of the liposome dispersion was applied on Formvar film-coated copper grids. Measurements were performed using a JEOL JEM 2100 HT (Jeol Ltd., Tokyo, Japan) transmission electron microscope under the accelerating voltage of 80 kV. Images of liposomes were taken using a 4 k × 4 k camera (TVIPS) equipped with EMMENU software ver. 4.0.9.87 (TVIPS GmbH, Gauting, Germany). 

#### 2.3.3. The Fluidity of the Vesicle Bilayer 

Fluorescence anisotropy assay using 1,6-diphenyl-1,3,5-hexatriene (DPH) as a probe was applied in order to characterize and compare the fluidity of the lipid bilayer of the anionic and cationic liposomes [38]. DPH in a methanol:chloroform (1:1, *v*/*v*) solution was introduced into the mixture of lipids dissolved in chloroform and liposomes were then prepared according to the previously described procedure. The samples were excited with vertically polarized light (λ = 350 nm) in order to measure the intensity of the light emitted perpendicular and parallel to the excitation beam at 428 nm. Measurements were performed using a Hitachi F-7100 spectrofluorometer. The following equation (Equation (1)) was used to calculate the fluorescence anisotropy (*r*):

(1) $$r=\frac{I_{VV}-{GI}_{VH}}{I_{VV}+{2GI}_{VH}},$$

where *G* is the correction factor (*G* = *I_HV_/I_HH_*); and *I_HV_* and *I_HH_* are the fluorescence intensities measured in the horizontal (H) or vertical (V) orientation of the emission beam polarizer in relation to the horizontally oriented polarizer of the excitation beam.

#### 2.3.4. Encapsulation Efficiency (*EE*) of GHK-Cu in Liposomes

The peptide encapsulation efficiency in liposomes was determined using the microdialysis method [39,40,41,42,43,44]. In order to separate the liposomes from the solution containing non-encapsulated actives, a regenerated cellulose membrane with a MWCO of 6–8 kDa (Spectra/Por) was used. The membrane was rinsed three times with distilled water and then immersed in a phosphate buffer (PBS) of pH = 6.00 for one hour to allow it to fully hydrate with the target medium. The samples of liposome dispersion (1 cm^3^) were poured into a dialysis bag and placed in a thermostatic dialysis chamber containing the PBS (100 cm^3^) for four hours at room temperature. The amount of unencapsulated GHK-Cu which diffused into the solution was determined via HPLC (Agilent 1100, Agilent Technologies, Inc., Santa Clara, CA, USA) using a UV-Vis detector at a wavelength of 220 nm using a 0.2% trifluoroacetic acid solution (TFA) and the ODS C18 column (Keystone Scientific, Inc. New Orleans, LA, USA). As a reference, a 0.5 mg/cm^3^ peptide solution in PBS was dialyzed to analyze the amount of GHK-Cu which could be transported across the membrane using the same conditions as for the liposome samples. *EE* was calculated using the following Equation (2):

(2) $$EE=\frac{TD-UD}{TD}\times 100\%$$

where *TD*—total peptide concentration; *UD*—unencapsulated peptide concentration.

#### 2.3.5. Release Kinetics of GHK-Cu Tripeptide from Liposomal Dispersions

The release study of GHK-Cu tripeptide was performed via the dialysis method according to our previous work [45]. An appropriate amount of liposome dispersions was placed in the dialysis bags which were sealed and put in the thermostated chambers filled with receptor solution (PBS, pH = 7.4) at 32 ± 0.5 °C. Sink conditions were provided during the dialysis. The samples were collected and analyzed for 24 h. The concentration of the released tripeptide in the receptor medium was analyzed using HPLC (details of the chromatographic analysis are described in Section 2.3.4). The amount of the released actives from the formulations was expressed as the ratio of the quantity of the released substance to the total amount of the incorporated compound as a function of time. Three mathematical models were used to analyze the obtained results: zero order [46], Higuchi [47,48] and Korsmeyer–Peppas [49,50].

### 2.4. Tyrosinase Inhibition Assay

To determine the influence of GHK-Cu on tyrosinase activity, the following protocol [51] was used. A solution containing 100 mM sodium phosphate of pH = 6.5, 2 mM L-DOPA and 0.5 mg/cm^3^ of aqueous GHK-Cu solution or water was used for the analysis for the peptide sample or reference sample, respectively. The concentration of the peptide solution was identical to that used in the liposomes. The reaction was started by adding 0.2 mg/cm^3^ of tyrosinase solution. The mushroom tyrosinase was used in the study. Changes in the absorbance associated with the formation of dopachrome were measured at 475 nm using a spectrophotometer (Nanocolor UV/Vis, MACHEREY-NAGEL GmbH & Co. KG, Dueren, Germany). The final result was calculated as an average of three measurements. Tyrosinase inhibition (*I_T_*) is expressed via the following Equation (3):

(3) $$I_{T}=\frac{AR-AS}{AR}\times 100\%,$$

where *AR*—absorbance of the reference sample: *AS*—absorbance of the peptide-containing sample. 

### 2.5. Elastase Inhibition Assay

In order to determine the effect of the GHK-Cu peptide on the inhibition of the elastin degradation process, activity tests against pancreatic porcine elastase were performed. Elastase from porcine pancreas was dissolved in a 0.1 M Tris-HCl buffer (pH = 8.00) to provide a working solution of 10 μg/mL. In a similar manner, a 1 mM solution of N-succinyl-Ala-Ala-Ala-p-nitroanilide (substrate) was obtained. Additionally, 25 μL of a 0.1 M Tris-HCl buffer, 25 μL of an elastase solution and 25 μL of a peptide or Tris-HCl solution (for reference assay) were added to each well of the plate. The mixture was incubated in the dark at 25 °C for 15 min. Then, 100 μL of substrate solution or 100 μL of Tris-HCl buffer was added. The samples were incubated for fifteen minutes at 37 °C. Measurements were made after thirty minutes using the Infinite F/M 200 Pro Nanoquant plate reader, measuring the absorbance at 405 nm. The peptide concentration was 0.5 mg/cm^3^. 

The percentage elastase inhibition was determined using the following Equation (4):

(4) $$I_{E}=\frac{\left(AR-AB\right)-(AS-ABS)}{AR-AB}\times 100\%,$$

where *AR*—absorbance of the reference sample; *AB*—absorbance of the blank; *AS*—absorbance of the peptide-containing sample; *ABS*—absorbance of the blank sample.

### 2.6. The Cytotoxicity Study

In order to evaluate the potential cytotoxicity of copper tripeptide in the “free” form and encapsulated in anionic liposomes, evaluation of cell viability was determined using an EpiDerm-200-reconstructed human epidermis model (RHE) [52]. The applied procedure was described in detail by Malinowska et al. [53]. Sodium dodecyl sulfate and phosphate buffer solution were used as a positive and negative control, respectively. The relative cells viability was calculated using the following Equation (5):

(5) $$RV=\frac{A_{TS}}{A_{NC}}\times 100\%,$$

where *A_TS_*—absorbance of the tested sample; *A_NC_*—absorbance of the negative control.

## 3. Results and Discussion

### 3.1. The Physicochemical Properties and Stability of Liposomes

In this work, liposomes based on hydrogenated lecithin as a source of phospholipids were prepared using a phosphate buffer of pH = 6.00 as the hydrating medium. This slightly acidic value was chosen in order to ensure the greater stability of the carriers in the formulation due to subsequent purpose of the liposome dispersion in cosmetic formulation with a pH suitable for human skin [54].

In order to determine the best lipid composition of the carriers for further testing, samples of cationic liposomes containing stearylamine as the charge-introducing agent at concentrations of 7%, 10% and 15% wt. and anionic liposomes containing dicetyl phosphate at concentrations of 5%, 7%, 10% and 15% wt. were prepared. The effects of SA and DCP concentrations on the properties of liposomes are presented in Table 2.

Large unilamellar vesicles (LUV) were obtained. The data presented in Table 2 show that all prepared liposomes were characterized by their sizes on the nanometric scale (oscillating around 100 nm) and very low polydispersity. The small size of the liposomes is advantageous in terms of improving transport across the epidermis and delivering the peptide to the deeper layers of the skin [55]. There was no important effect of SA and DCP concentration on the diameter of the liposomes; however, the cationic liposomes were slightly larger than the anionic liposomes (by around 30 nm). This phenomenon was also observed by Z. Németh et al. [56]. X. Wang et al. described GHK-Cu liposomes prepared using a similar procedure resulting in 117.2 nm vesicles and high homogeneity [35]. The size of the liposomes affects their ability to transport the active ingredient into the skin [57]. The use of the extrusion method through a 100-nm membrane allowed us to obtain a dispersion with small particle sizes [34].

**Figure 1:**
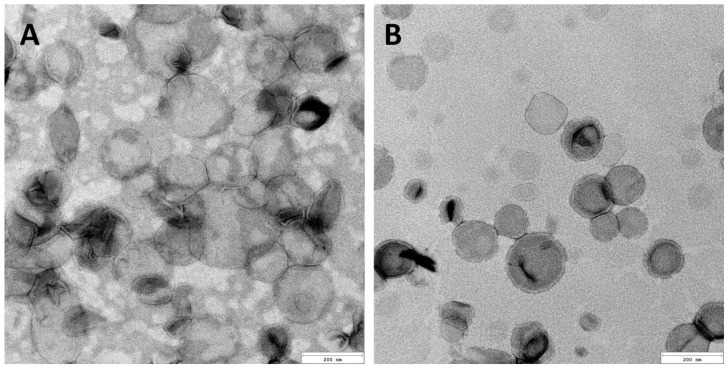
TEM images of anionic (**A**) and cationic (**B**) liposomes. The bar represents 200 nm.

The TEM imaging (Figure 1) of the prepared carriers showed that they represent the shape of nanospheres with dimensions corresponding to DLS measurements (Table 2). There is no significant difference between the liposomes obtained through the two techniques. A set of homogeneous particles can be observed in the case of the application of both methods. Moreover, it can be seen that the morphology of cationic and anionic liposomes is congenial. 

The obtained results confirm that the introduction of a charge onto the surface of the liposomes, which causes electrostatic repulsion, increases the stability of the system and prevents the aggregation of the dispersed particles. The results of measuring the liposome zeta potential prove their stability (the value of ±30 mV is considered sufficient to ensure the dispersion stability [58,59]). As shown in Table 2, the zeta potential decreased from −25.3 ± 0.5 mV for 5% DCP to −40.5 ± 2.1 mV for 15% DCP with increasing DCP concentration. In the case of the cationic liposomes, the increase in SA concentration caused only a slight decrease in the value of the zeta potential from 48.8 ± 1.9 mV to 42.3 ± 1.8 mV for 7 and 15% SA, respectively. The samples with the highest absolute zeta potential values (with highest stability), i.e., those containing 15% DCP for anionic liposomes and 7% SA for cationic carriers, were selected for further tests.

Amino acids and peptides, due to their amphoteric nature, can influence the physicochemical parameters of the carriers in which they are encapsulated, thus changing their stability [60]. Table 3 shows the effect of the tripeptide concentration on the properties of the liposomes. The concentration of GHK-Cu in the PBS used for lipid film hydration was 1, 5 and 10 mg/cm^3^. It was observed that with the increase in the peptide concentration, the absolute value of the zeta potential decreased for both anionic and cationic liposomes, but no significant influence of the concentration of the active substance on the size of the liposomes was noted. It is probably caused by the extrusion process. The pore size of the membrane used was 100 nm. The best samples in terms of the appropriate (greater than ±30 mV) zeta potential value were obtained in the case of the lipid film hydration with a solution containing 5 mg/cm^3^ of GHK-Cu for both types of liposomes. The use of a higher concentration of the active compound may shift the equilibrium state of the encapsulation process and increase its total amount inside the carriers; however, further tests of liposomes hydrated with a peptide solution of a higher concentration (10 mg/cm^3^) were abandoned due to their low zeta potential value (−28.4 ± 1.2 mV).

**Figure 2:**
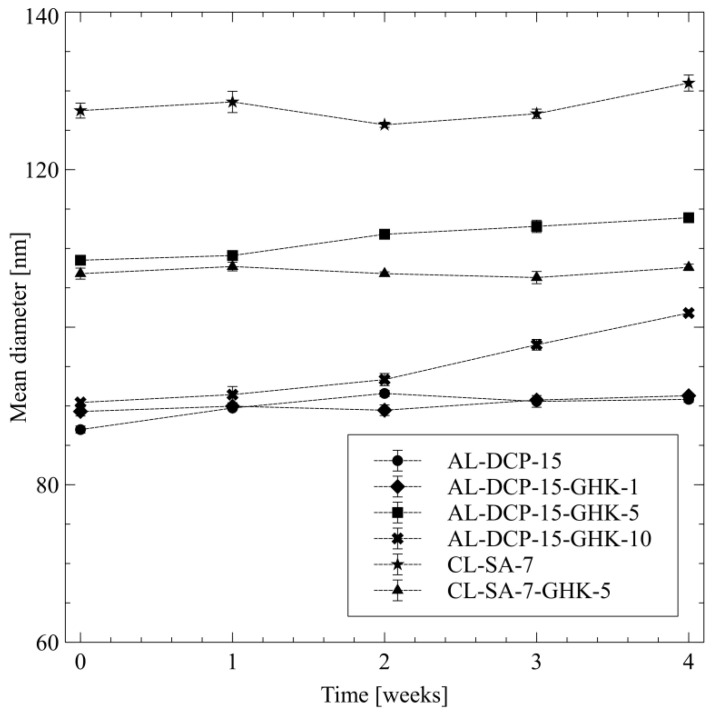
The stability of the prepared liposomes over four weeks expressed as the change of their mean diameter ± SD, n = 3.

The stability of the copper tripeptide-loaded and unloaded carriers was evaluated by monitoring the size and zeta potential over the course of four weeks. Low values of polydispersity index (PDI) and high absolute values of zeta potential suggest that the liposomes are stable and do not undergo aggregation (Table 3). It was observed that during storage time, in the cases of both anionic and cationic vesicles, there were no significant differences in the average vesicle sizes; only in the case of the AL-DCP-15-GHK-10 was there a gradual, steady increase in liposomes diameter, which was probably an effect of the aggregation (Figure 2). In this case, the value of the zeta potential (−28.4 ± 1.2 mV) also indicates the low stability of the sample.

The fluidity of the lipid bilayer is an important parameter of liposomes, which affects the release of active substances from the phospholipid systems and the ability to retain them inside the carrier as well as the skin permeation [61,62]. The steady-state fluorescence anisotropy measurements results, using DPH as the fluorescent probe, are presented in Table 4. 

It is evident that anionic liposomes (AL-DCP-15) were characterized by an almost three times higher value of fluorescence anisotropy (0.165) than in the case of the cationic liposomes (0.059). This indicates greater stiffness of the lipid bilayer of anionic liposomes [63,64,65]. This may be due to the stronger effect of increasing the phase transition temperature in the case of DCP than in SA [66,67].

### 3.2. Encapsulation Efficiency of GHK-Cu in Liposomes

Due to their unique structure, liposomes are potential carriers for peptides; however, achieving very high *EE* is difficult. Both liposome concentration and the affinity of the peptide to the core of the carriers influence the efficiency of the encapsulation. In the study, different lipid compositions and concentration levels were used to achieve the highest GHK-Cu encapsulation. Additionally, to increase copper tripeptide *EE* in the liposome systems, five freeze–thaw cycles in liquid nitrogen were used as a step in the TFH/extrusion preparation method. This technique allows the actives to diffuse into the liposome structure by destroying the phospholipid bilayer [68,69,70,71,72,73].

The results of the study of GHK-Cu encapsulation efficiency in the prepared liposome systems are presented in Table 5. The data show the effect of the total lipid composition and the cooper tripeptide concentration in the hydrating solution on the *EE*.

In the case of anionic liposomes, for the peptide concentration of 0.5 mg/cm^3^, an almost twofold increase was observed in the encapsulation efficiency from 17.3% to 31.7% with an increase in the lipid concentration from 10 to 25 mg/cm^3^. This corresponds with the number of liposomes formed and thus the space available for entrapment of the active ingredient. This aspect is dominant; the encapsulation volume of the liposomes is practically identical in all samples because of their size homogeneity. However, further increasing the lipid concentration to 50 mg/cm^3^ did not bring the expected increases in *EE*, which is observed as a plateau. These results are in agreement with the results of X. Xu et al. [71]. At a high concentration of lipids, the liposome dispersion became a highly viscous jelly-like form preventing the peptide solution from flowing into the interior of the carrier. In the case of cationic liposomes, with a lipid concentration of 25 mg/cm^3^, an *EE* of 20.0 ± 2.8% was obtained. This is a significant, approx. 1.6-fold reduction in the *EE* value in relation to the anionic liposomes. The observed phenomenon can be explained by the different strength of interaction between the oppositely charged types of carriers and the active substance under the given conditions. The isoelectric point value (pI) is an important parameter for amphoteric substances (like peptides). The value of pI for GHK-Cu (10.06) was calculated using the online calculator developed by Kozlowski [74]. At pH = 6.00, the peptide is in the cationic form and is probably more attracted to liposomes of an anionic nature, thus increasing the *EE*. The higher *EE* value may therefore also be the result of the additional adsorption of the peptide on the surface of the nanoparticle and not only its presence in the liposome core [75]. This also explains the lower *EE* values for the cationic liposomes. Due to this effect, it is also possible to obtain higher concentrations of the peptide inside the electrically charged carrier than in the case of neutral liposomes, where there would be no interaction favoring encapsulation [76,77,78]. S. Erdem et al. [34] using DPPC or soy lecithin and cholesterol, obtained similar efficiencies of copper tripeptide encapsulation in liposomes at levels of 27% and 33%, respectively. However, the ratio of total lipid concentration (10 mg/cm^3^) to peptide concentration (0.06 mg/cm^3^) is approximately 3.3 times higher than that of our liposomes, meaning they are less efficient at encapsulating the same amount of active ingredient than the carriers obtained in this work. The reason for this may be an additional freeze–thaw step, increasing *EE* and the use of extrusion, as well as the uniformity of vesicle sizes.

Increasing the concentration of the tripeptide in the hydrating medium from 0.5 to 5 mg/cm^3^ resulted in a significant 1.8- and 2.9-fold decrease in the encapsulation efficiency for anionic and cationic liposomes, respectively. This may be explained by the equilibrium between the peptide concentration inside and outside of the liposomes. However, at the same time, the absolute amount of GHK-Cu in a unit volume of the liposomal dispersion is about 5.5 and 3.5 times higher for anionic and cationic carriers, respectively, which is the result of an unequal *EE* change in response to the total peptide concentration. An example of this is the fact that for anionic liposomes, a 10-fold increase in the peptide concentration causes only about a 1.8-fold decrease in *EE*. Thus, in order to ensure the highest efficiency of a peptide in a cosmetic formulation, using carriers with a lipid concentration of 25 mg/cm^3^ hydrated with 0.5 mg/cm^3^ GHK-Cu solution is preferred. According to research [79], the increase in the production of collagen and elastin is achieved using significantly lower GHK-Cu concentrations (0.01–100 nM), which means that an encapsulation efficiency of approximately 32% should be sufficient to obtain the desired effect. Higher concentrations of the peptide, despite the greater absolute amount encapsulated inside the carriers, may not be economically justified because of significant amounts simultaneously remaining outside the liposomes, and thus potentially not penetrating the hydrophobic epidermis. However, lower (10 mg/cm^3^) concentrations of lipids do not give satisfactory *EE* values, and higher concentrations (50 mg/cm^3^) do not lead to beneficial changes.

Taking into account the incomplete diffusion of the GHK-Cu peptide (93.40 ± 0.96%) through the cellulose membrane, the obtained *EE* results were corrected by this factor. A proportion of the peptide is likely to be absorbed into the surface or channels of the cellulose membrane because of its affinity for hydrophilic film. A similar phenomenon has already been reported [80,81,82,83].

### 3.3. Release Kinetics of GHK-Cu Tripeptide from Liposomal Dispersions

**Figure 3:**
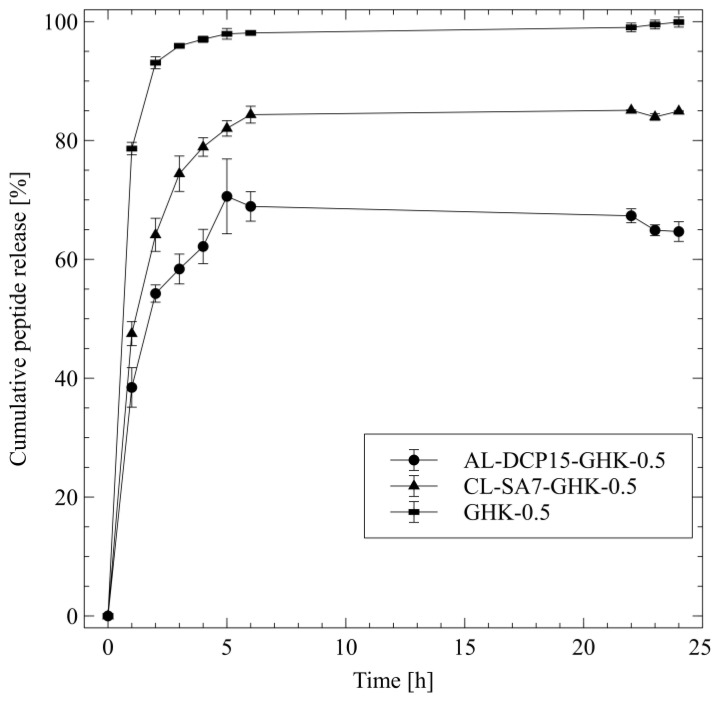
The release profiles of GHK-Cu from different carriers, expressed as cumulative peptide release [%] ± SD, n = 3.

The study of active substances’ release kinetics allows us to better understand their efficacy, which is important, especially in the case of cosmetic or pharmaceutical products. Figure 3 shows the release profiles of the GHK-Cu peptide from anionic (AL-DCP15-GHK-0.5) and cationic (CL-SA7-GHK-0.5) liposome dispersions and from the PBS solution (GHK-0.5).

The obtained results show that after about 4 h, almost 100% of GHK-Cu was released from the reference sample (tripeptide PBS solution), and the diffusion itself was characterized by a rapid shape, reaching a value of 99.9 ± 0.8% after 24 h. The release amount of the peptide form liposomes was lower: 84.9 ± 0.1% and 64.7 ± 1.7% for cationic and anionic phospholipid carriers, respectively. The higher value of the cooper tripeptide release from cationic liposomes is probably related to the higher fluidity of the lipid bilayer, which may affect the migration of the active substance [84].

The selection of a suitable kinetic model for fitting the GHK-Cu release data helps in determining the release characteristics. There are a number of kinetic models, which describe the overall release of the drug from the carrier. The release of cooper peptide was investigated using three mathematical models: zero order model (Equation (6)), Higuchi model (Equation (7)) and Korsmeyer–Peppas (Equation (8)).

(6) $$\frac{M}{M_{0}}=K_{0}\times t$$

(7) $$\frac{M}{M_{0}}=K_{H}\times t^{0.5}$$

(8) $$\frac{M}{M_{0}}=K_{KP}\times t^{n}$$

where *M/M*_0_—mass fraction of the substance released; *t*—time; *K*_0_*, K_H_, K_KP_*—zero order, Higuchi and Korsmeyer–Peppas rate constant; *n*—diffusional exponent.

The results of kinetic analysis are shown in Table 6.

It can be observed that none of the formulations had good agreement with the zero-order kinetics model, which indicates that the concentration of the permeating ingredient is not uniform over time, yet rather shows an increased release rate at the beginning of dialysis. Since the peptide solution in PBS does not contain any carriers, it cannot be described in terms of release according to the Korsmeyer–Peppas (K-P) model, and the diffusion itself is probably limited by the viscosity of the medium.

In the case of liposomes, the K-P model showed the best fit for both samples (R^2^ of 0.943 and 0.973 for AL and CL, respectively). The characteristic parameter *n*, appearing in the equation, allows us to determine the type of diffusion. For AL-DCP15-GHK-0.5, it reached a value of 0.511, which indicates an anomalous, non-Fickian transport [50]. The diffusion of the tripeptide from CL-SA7-GHK-0.5 was based on the Fickian release mechanism [85].

### 3.4. Enzyme Inhibition Assays

In order to further investigate the potential cosmetic applications of the GHK-Cu peptide as a whitening and anti-aging agent, mushroom tyrosinase and elastase activity tests were conducted. Tyrosinase is the enzyme responsible for the formation of melanin in the skin, enclosing two copper atoms surrounded by protein histidine units in the active centre [86]. For this reason, the GHK-Cu tripeptide containing histidine in its structure, and additionally complexing copper ions could be suspected of causing tyrosinase inhibition. Elastase is one of the enzymes found in the skin and is responsible for the degradation of elastin. It is a structural protein with high flexibility, responsible for, among other effects, the ability of the skin to return to its original state after applying pressure. An increase in elastase activity may result in a decrease in skin elasticity and the formation of wrinkles [87].

The results of tyrosinase and elastase activity assay is shown in Table 7.

Despite there being a fairly high concentration of the active ingredient, the degree of tyrosinase inhibition (10.23 ± 3.03%) by the peptide was negligible. It can therefore be concluded that GHK-Cu is not an effective tyrosinase inhibitor. Nevertheless, the GHK-Cu peptide can be used as a supporting substance in whitening products. It shows an ability to protect the skin against oxidative stress and cells against UV radiation as well as reducing inflammation, including modifying the expression of metalloproteinases [6,7]. However, GHK-Cu used at a concentration identical to that in liposomes (0.5 mg/cm^3^) deactivated elastase by almost 50% (Table 7), thus displaying good enzyme inhibitory properties [88]. This may be attributed to an increase in skin elasticity by inhibiting the activity of the enzyme which breaks down elastin, opening new paths for using the ingredient in dermocosmetics. Additionally, the copper contained in the peptide can also modify the activity of lysyl oxidase, which is necessary for increasing the synthesis of collagen and elastin and structurally supports the skin and reduces wrinkles [89,90].

### 3.5. Study of GHK-Cu Cytotoxicity

Cosmetic raw materials should be safe and cannot show any cytotoxic potential to the skin cells. In order to assess the safety of GHK-Cu tripeptide, both in PBS solution and encapsulated in anionic liposomes, the EpiDerm-200-reconstructed human epidermis cells model were used. Table 8 presents the obtained results for 0.5 mg/cm^3^ GHK-Cu, used as a reference sample, AL-DCP15-GHK-0.5 and for the positive (PC) and negative (NC) controls.

As anionic liposomes show a lower tendency to cytotoxicity than cationic carriers [91,92], only anionic samples were tested. The obtained data confirmed that the prepared anionic carriers are safe and do not exhibit toxic properties on the cells (96.6% cells viability) yet showed that the GHK-Cu-loaded liposomes do not increase the human cells proliferation (values lower than NC). In comparison to liposomes, the peptide solution shows slightly lower cell viability values (93.1%), which indicates that the peptide encapsulation in liposomes can be also beneficial in terms of increasing the GHK-Cu peptide bioavailability. 

## 4. Conclusions

The stable anionic and cationic GHK-Cu-loaded (0.5 mg/cm^3^) liposome systems of small sizes (approx. 100 nm) were obtained using the thin-film hydration method combined with freeze–thaw and extrusion techniques. Stearylamine (SA) and dicetyl phosphate (DCP) were selected as the charge introducing agents. The obtained results proved that the lipid composition (quality and quantity) influences both the liposomes properties and their stability. The addition of SA or DCP into the liposome lipid bilayer affects vesicle flexibility and preserves their integrity. Fluorescence anisotropy tests indicated that bilayer fluidity was higher in the case of cationic liposomes (CL-SA-7). On the other hand, anionic liposomes (AL-DCP-15) were characterized by lower lipid bilayer fluidity, which may be the effect of higher phase transition temperatures of dicetyl phosphate than stearylamine. Moreover, the charge modification of the liposome surface influences the encapsulation efficiency of the GHK-Cu. The anionic liposomes showed higher encapsulation efficiency of the cooper tripeptide than the cationic liposomes, which is probably due to the additional electrostatic interaction between the positively charged peptide and the negatively charged surface of the carrier. The obtained results also indicated that a lipid concentration of up to 25 mg/cm^3^ influences the increase in the peptide *EE*, which corresponds with the number of available vesicles. The release studies proved that the mechanism of the peptide release from liposomes followed the Fickian diffusion for cationic and anomalous, non-Fickian mechanism for anionic carriers. Moreover, anionic liposomes, selected as the best sample, were characterized by compatibility with the peptide and were not cytotoxic towards RHE cells.

The results of the inhibition of elastase and tyrosinase tests conducted in order to predict GHK-Cu activity and further application in cosmetic formulations show that a peptide in concentration of 0.5 mg/cm^3^ insignificantly affects the mushroom tyrosinase but leads to high elastase inhibition (48.90 ± 2.50%), thus reducing the rate of elastin degeneration and supporting the structural integrity of the skin.

To summarize, the charged liposomes can be applied as carriers for biomimetic peptides such as copper-binding peptide and used as potential active ingredients in anti-aging cosmetics. Nevertheless, taking into account the liposomes’ safety of use, the anionic ones have greater application potential. 

## Figures and Tables

**Table 1:** Anionic and cationic liposome compositions. The lecithin to cholesterol weight ratio was 14:1 in all samples.

Sample	Concentration [% wt.]	
	DCP	SA
AL-DCP-5	5	-
AL-DCP-7	7	-
AL-DCP-10	10	-
AL-DCP-15	15	-
CL-SA-7	-	7
CL-SA-10	-	10
CL-SA-15	-	15

**Table 2:** The effects of SA and DCP concentrations on the properties of liposomes (n = 3).

Sample	DCP [% wt.]	SA [% wt.]	Mean Diameter ± SD [nm]	Zeta Potential ± SD [mV]	PDI ± SD
AL-DCP-5	5	-	92 ± 0	−25.3 ± 0.5	0.038 ± 0.012
AL-DCP-7	7	-	96 ± 0	−31.5 ± 1.5	0.041 ± 0.005
AL-DCP-10	10	-	102 ± 1	−34.8 ± 1.1	0.060 ± 0.015
AL-DCP-15	15	-	87 ± 0	−40.5 ± 2.1	0.080 ± 0.011
CL-SA-7	-	7	128 ± 1	48.8 ± 1.9	0.139 ± 0.014
CL-SA-10	-	10	131 ± 0	45.5 ± 2.8	0.056 ± 0.006
CL-SA-15	-	15	120 ± 1	42.3 ± 1.8	0.111 ± 0.014

**Table 3:** The effect of GHK-Cu tripeptide concentration on the properties of liposomes (n = 3).

Sample	GHK-Cu Concentration [mg/cm^3^]	Mean Diameter ± SD [nm]	Zeta Potential ± SD [mV]	PDI ± SD
AL-DCP-15	-	87 ± 0	−40.5 ± 2.1	0.08 ± 0.011
AL-DCP-15-GHK-1	1	89 ± 1	−37.9 ± 1.4	0.087 ± 0.014
AL-DCP-15-GHK-5	5	109 ± 0	−38.1 ± 4.7	0.075 ± 0.017
AL-DCP-15-GHK-10	10	91 ± 0	−28.4 ± 1.2	0.084 ± 0.009
CL-SA-7	-	128 ± 1	48.8 ± 1.9	0.139 ± 0.014
CL-SA-7-GHK-5	5	107 ± 1	36.9 ± 0.7	0.082 ± 0.027

**Table 4:** Fluorescence anisotropy test results for unloaded anionic and cationic liposomes (*G* = 1.350).

Sample	Fluorescence Anisotropy
AL-DCP-15	0.165
CL-SA-7	0.059

**Table 5:** Encapsulation efficiency results for anionic (AL) and cationic (CL) liposomes with different lipid and peptide concentrations.

Sample	GHK-Cu Concentration [mg/cm^3^]	Encapsulation Efficiency ± SD [%]		
		Total Lipid Concentration		
		10 mg/cm^3^	25 mg/cm^3^	50 mg/cm^3^
AL-DCP15-GHK-0.5	0.5	17.3 ± 0.6	31.7 ± 0.9	33.4 ± 0.9
AL-DCP15-GHK-5	5	-	17.5 ± 1.3	-
CL-SA7-GHK-0.5	0.5	15.7 ± 0.7	20.0 ± 2.8	-
CL-SA7-GHK-5	5	-	6.9 ± 1.1	-

**Table 6:** The parameters of selected kinetic models for tested samples.

Kinetic Model	Parameter	Sample		
		AL-DCP15-GHK-0.5	CL-SA7-GHK-0.5	GHK-0.5
Zero-order	R^2^	0.513	0.490	0
	*K*_0_·10^1^ [h^−1^]	1.46	1.79	2.21
Higuchi	R^2^	0.938	0.934	0.718
	*K_H_*·10^1^ [h^−0.5^]	3.17	3.88	4.90
Korsmeyer–Peppas	R^2^	0.943	0.973	0.823
	*K_KP_*·10^1^ [h^−n^]	2.84	13.2	7.18
	*n*	0.511	0.190	0.318

**Table 7:** Inhibitory activity of the GHK-Cu peptide against the enzymes used in the assay—tyrosinase and elastase.

Degree of Inhibition [%] ± SD	
Tyrosinase	Elastase
10.23 ± 3.03	48.90 ± 2.50

**Table 8:** Viability assay of the RHE cells after exposure to tested samples.

Sample	Viability [%] ± SD
GHK-Cu-0.5	93.1 ± 11.0
AL-DCP15-GHK-0.5	96.6 ± 6.1
NC	100.0 ± 0.8
PC	6.2 ± 0.6

## Data Availability

The data that support the findings of this study are contained within the article.
